# Longitudinal study of cognitive and mental functions among adult Hodgkin-lymphoma survivors, based on data from a primary treatment center in Hungary

**DOI:** 10.3389/fonc.2025.1509424

**Published:** 2025-02-17

**Authors:** István Virga, Karolina Kósa, Anna Illés, Zsófia Miltényi, Tibor Ivánka, Roland Berecz, Anikó Égerházi, Árpád Illés, Ferenc Magyari

**Affiliations:** ^1^ Division of Haematology, Department of Internal Medicine, University of Debrecen, Debrecen, Hungary; ^2^ Doctoral School of Clinical Medicine, University of Debrecen, Debrecen, Hungary; ^3^ Department of Anesthesiology and Intensive Care, University of Debrecen, Debrecen, Hungary; ^4^ Department of Behavioral Sciences, University of Debrecen, Debrecen, Hungary; ^5^ Department of Psychiatry, Faculty of Medicine, University of Debrecen, Debrecen, Hungary

**Keywords:** Hodgkin-lymphoma, cancer-related cognitive impairment (CRCI), computerized neuropsychological test battery (CANTAB), health-related quality of life, chemobrain

## Abstract

**Introduction:**

Due to risk and response-adapted treatment strategies, more than 80% of newly diagnosed adult classical Hodgkin lymphoma (HL) patients at any stage can be cured and become long-term survivors. A well-known side effect is cognitive dysfunction that appears in HL patients after chemotherapy (chemobrain). In the present longitudinal study, we measured cognitive function in our HL patients, in search of potential correlations between patient-related factors, the signs and symptoms of their diseases, and therapeutic factors.

**Methods:**

Patients underwent a computer-assisted assessment (CANTAB) of cognitive function (especially domains of visual memory, attention, working memory, and planning) and filled out psychological questionnaires (standardized, self-administered and validated for Hungarian language) before treatment (n=30, T1) and after the first-line treatment (n=25, T2), and 8.6 years after the end of chemotherapy (n=19, T3).

**Results:**

The median age of 16 females and 14 males was 35 years (20-69), 35 years (21-63) after chemotherapy, and 43 years (29-70) at the end of the long-term follow-up, when the study was completed. 77% of all patients showed cognitive impairment before treatment. A close correlation was found between attention and unfavorable prognostic factors (III-IV. stage, age, bulky) baseline comorbidities (T2DM, psoriasis, HTN) and place of residence. Visual memory was affected by comorbidities and the place of residence. Working memory and planning was influenced by single marital status, and bulk disease. Post-treatment cognitive impairment was evaluated in 77% of the HL patients. In the working memory and planning domain, the Stockings of Cambridge (SOC) subtest significantly improved after treatment, while visual memory and attention remained unchanged. The cumulative dose of bleomycin associated with SOC.

**Conclusion:**

The study highlights the fact that cognitive functions of HL patients were already impaired before treatment, especially attention, working memory, and planning. Long-term improvement in cognitive function was observed post-treatment. Employment status, place of residence and unfavorable prognosis have an impact on cognitive domains. Early diagnosis and intervention are essential to maintain patients’ quality of life throughout and after treatment.

## 1 Introduction

Thanks to modern risk- and response-adapted treatment strategies, more than 80% of newly diagnosed Hodgkin-lymphoma (HL) patients at any stage can be cured and are expected to be long-term survivors (1, 2). In 2020, 83.087 new HL patients were diagnosed worldwide; thus, HL accounted for 0.4% of all cancer cases (3). The incidence rate is 2-3/100.000 inhabitants in Hungary, which means approximately 2-300 newly diagnosed cases each year (4, 5). HL primarily affects active young adults: a significant portion of patients were working-age young adults at the time of diagnosis. Therefore, the social and economic implications of the disease outweigh its incidence rate. The long-term survival rate is mainly decreased by developing second malignancies and the appearance of organ damage (heart, lungs, thyroid gland, etc.). Therefore, treatment-related side effects must be focused on, but health-related quality of life and patients’ return to work after successful treatment should also be considered (6). Thanks to favorable treatment results, survivorship quality is becoming more prominent nowadays. Cancer-related cognitive impairment (CRCI) is a relevant adverse effect experienced by patients during and after chemotherapy (chemobrain) for non-central nervous system cancer/lymphoma (7). Many factors could influence CRCI. Factors not related to chemotherapy related factors include age, gender, education, depression, psychological stress and fatigue. Treatment-related factors include the type of chemotherapy and its direct and indirect neurotoxic effects, which can occur even when the blood-brain barrier is intact. In addition, the type of disease must also be considered, as not all hematological disorders require immediate treatment (indolent malignancies) (8–10). CRCI mainly affects the functions of the domains of visual memory, attention, working memory and planning (9, 11, 12). The complexity of cognitive assessment in cancer survivors is unique due to the two distinct aspects of cancer-related cognitive impairment (CRCI): the cognitive symptoms reported by the survivors themselves (subjective) and the cognitive changes observed through formal neuropsychological testing (objective) (13). According to the literature data, there is a weak or absent association between objective neurocognitive procedures and self-reported cognitive test results (14). In our article, we used only objective neurocognitive tests and mental health questionnaires to characterize the cognitive performance of our HL population. There are few published data available on cured HL patients (8, 15–18). Unfortunately, longitudinal studies (with pretreatment measurement) of cognitive function have been severely lacking among patients with HL. Only three prospective longitudinal trials were performed on repeated measurements of cognitive functions among HL patients. Janelsins et al. performed a nationwide study that included a high number of patients with lymphoma who were significantly worse over time on objective assessments of cognitive function compared with sex- and age-paired participants serving as noncancer controls assessed at the same time intervals as patients (19). Dan Fayette et al. reported that the presence of cognitive impairment in HL patients appeared before the treatment and increased damages caused by chemotherapy (20). Jurickova et al. highlighted that cognitive deterioration can appear before the initiation of treatment, independently of anxiety, depression or physical symptoms (21).

Although there are studies of pediatric and adolescent Hodgkin lymphoma survivors with long-term neurocognitive impairment (attention, memory, executive function), the big difference is that they are treated during critical periods of growth and development. However, the role of neurotoxic treatment, level of inflammation and oxidative stress, and secondary neurocognitive decline related to other organ (cardiovascular, pulmonary) damage can also be highlighted here (22).

Our longitudinal study aimed at the follow-up of cognitive dysfunction in newly (treatment-naive) diagnosed HL patients before and immediately after treatment completion as well as after several years in order to find potential correlations between patient-related factors, specific signs and symptoms of their diseases, and therapeutic factors.

## 2 Materials and methods

### 2.1 Subjects and data collection

**Figure 1:**
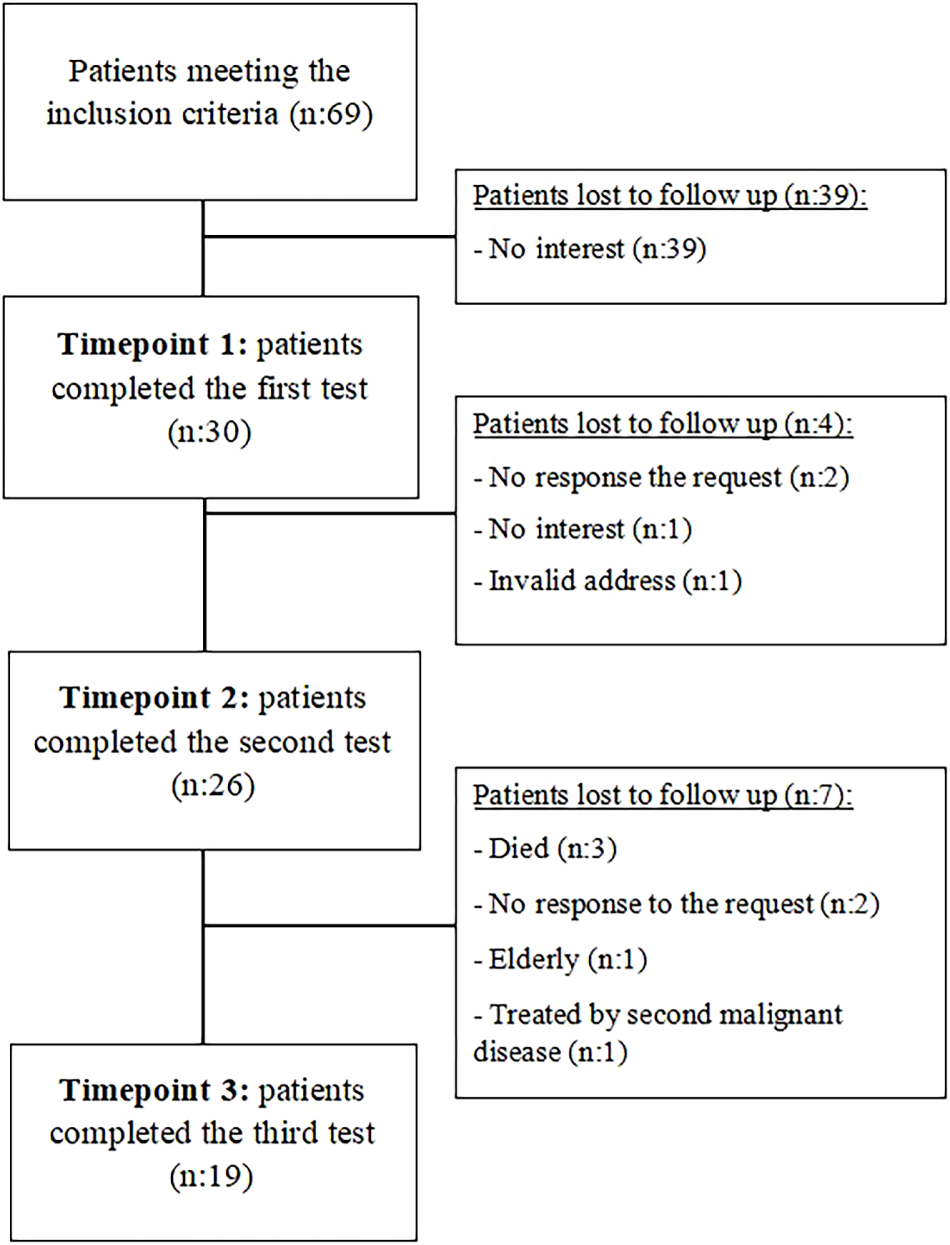
Flowchart of the follow-up of Hodgkin-lymphoma patients.

A single-site, single-arm longitudinal survey was conducted at the Division of Hematology of the University of Debrecen. Altogether, 30 adult treatment-naive HL patients were recruited between December 13, 2012, and January 31, 2016. Exclusion criteria were lack of signed informed consent, age under 18 years at the time of diagnosis, the presence of central and peripheral nervous system involvement with HL, and any previous cancer treatment. Age, gender distribution, clinical stage and type of treatment of the selected patients were no different from that of our population of HL patients (5) **Supplementary Table 1**. Based on the hospital records of the patients, other diseases diagnosed before the initiation of HL treatment were defined as comorbidities, whereas treatment-related side effects were defined as conditions diagnosed in the follow-up phase after HL treatment. Between 2013-2016, we treated 69 HL patients in our clinic, 30 of whom consented to the study. The flow diagram of the participants is presented in **Figure 1**.

### 2.2 Treatment protocols

Hungarian guidelines recommend ABVD (adriamycin 25 mg/m2, bleomycin 10 mg/m2, vinblastin 6 mg/m2, and dacarbazin 375 mg/m2) protocol for HL. One ABVD cycle consists of two treatment days in a 28-day period (all doses given on days 1 and 15). Patients with favorable early-stage disease (stage I-II) receive 2-4 cycles ABVD, those with unfavorable prognosis receive 4-6 cycles of ABVD and radiotherapy (if it is necessary). Six cycles of ABVD chemotherapy alone are required for advanced-stage disease (stage III-IV). For patients (n=8) with unfavorable prognosis in advanced stage (stage III/IV of HL) the BV+AVD (brentuximab vedotin 1,2mg/m2, doxorubicin 25 mg/m2, vinblastin 6mg/m2, dacarbazin 375 mg/m2) protocol can be used at first line. Treatment plan consists of 6 chemotherapy cycles. Each cycle lasts 4 weeks (28 days). For each cycle, two treatments are given 2 weeks apart (Day 1 and Day 15) (23). The response for the treatment was monitored by ^18^FDG-PET/CT. Involved-field/node radiotherapy was used after 1998 (20-30 Gy), respectively.

### 2.3 Study design and assessment

Subjects were asked to perform a series of 13 computerized neuropsychological tests of the Cambridge Neuropsychological Test Automated Battery (CANTAB, Cambridge Cognition, Cambridge, United Kingdom). CANTAB has been proven to be useful to assess cognitive functions in various neurological and psychiatric disorders (24, 25). Subjects were seated at a table at comfortable height, approximately 0.5 meters from the monitor, and were instructed to carry out the tasks by touching the screen. After an initial explanation and completing a simple *Motor Screening Task (MOT)* successfully (touching the center point of flashing crosses on the screen), subjects were given various tests in the following order (the technical description of the tests can be found on the Cambridge Cognition’s website: http://www.cantab.com):

*Big Little Circle (BLC):* a two-stimuli visual discrimination and category achievement test.

*Spatial working memory (SWM):* this task assesses the subject’s ability to retain spatial information and manipulate remembered items in working memory.

*Reaction time (RTI):* The task is designed to measure the subject’s speed of response to a visual target where the stimulus is either predictable (simple reaction time) or unpredictable (choice reaction time). A manual switch is used for the task.

*Spatial* sp*an (SSP):* A computerized version of the Corsi blocks, a test of span for spatial items similar to ‘digit span’ tests for verbal items.

*Pattern recognition memory (PRM):* An examination of visual recognition memory in a 2-choice forced discrimination paradigm.

*Spatial recognition memory (SRM):* This task tests visual-spatial memory in a 2-choice forced discrimination paradigm.

*Paired associate learning (PAL):* Assessment of simple visual pattern and visuospatial associative learning, which contains aspects of a delayed response procedure and a conditional learning task. Successful performance in the PAL test requires both the elaboration of “frontal strategies” and the “mnemonic processes” of the medial temporal lobe. They should pay attention to both the stimuli and their spatial position.

*Intra/Extradimensional shift task (IED):* A test of rule acquisition and reversal, featuring visual discrimination and attentional set-shifting and analogous to a category change in the Wisconsin Card Sorting Test.

*Match to sample visual search (MTS):* A two-stimuli visual discrimination and category achievement test.

*Delayed matching to sample (DMS):* This task tests visual memory in a 4-choice delayed recognition memory paradigm.

*Stockings of Cambridge (SOC):* The task is analogous to the ‘Tower of London’ test and assesses the subject’s ability to engage in spatial problem-solving. This test makes substantial demands on executive function.

*Rapid Visual Information Processing (RVP):* It is a visual continuous performance task, using digits rather than letters.

Results were compared to the internal normative database of CANTAB, involving 3.000 healthy volunteers, and were matched for age-groups and gender. CANTAB tests were previously validated among healthy Hungarian volunteers showing no statistically significant differences in cognitive performance compared to the internal normative database (24, 26).

The above listed tests examined the domains of visual memory (PAL, DMS, SRM), functions of attention (IED, RTI, RVP, PRM), working memory, and planning (SSP, SWM, SOC), although the CANTAB battery emphasizes assessment of frontostriatal functions (SWM, IED, SOC, and SWP). Some tests are sensitive to temporal lobe function (PAL, DMS, PRM, SSP) (27). Due to financial problems, RTI psychomotor/reaction time, SOC, SRM and MTS were not measured at time point 3.

### 2.4 Questionnaires

HL survivors were asked to complete a standardized, self-administered and validated Hungarian questionnaire, which included items on socio-demographic status (place of residence, marital status, educational level, employment, important life events after lymphoma treatment) and psychiatric treatment (date and type of medication) at the time of diagnosis, as well as the scales listed below. Data on the disease and its treatment were based on hospital records.

The Hungarian version of the Hospital Anxiety and Depression Scale (HADS-14) has been used in studies of distress among cancer patients in general. Each of the 14 items is scored on a 4-point scale (0–3). Sum scores for the anxiety and depression subscales are calculated by simple addition. Two different cut-off scores (8 or higher or 11 or higher on either scale) can be used for case definition. In this study, we used the lower cut-off for defining a case (28, 29).

The General Health Questionnaire (GHQ-12) is the most extensively used screening instrument for common mental disorders, in addition to being a general measure of psychiatric well-being. Each item is scored on a 4-point scale (corresponding to a symptom missing: ‘not at all’, ‘same as usual’, or present: ‘rather more than usual, or ‘much more than usual’). It can be scored in a bimodal fashion (0–0–1-1), when final scores range from 0 to 12. According to this method, patients scoring at or above five are considered at risk of severe mental distress (30).

The validated Hungarian version of the abbreviated Sense of Coherence (SOC-13) scale was used in the present survey to measure the overall capacity to cope with stressful situations. All 13 items are answerable on a Likert scale from 1 to 7, and total scores vary between 13 and 91. A higher score indicates a stronger SOC-13 (30).

The Perceived Stress Scale 4 (PSS-4) is the most widely used psychological instrument for measuring stress perception. Scores for the 4-item form range from 0 to 16. Potential responses range from 0 (never) to 4 (very often), and positively stated items are reverse coded before items are summed up with higher scores indicating more perceived stress (31).

The Dysfunctional Attitude Scale form A (DAS-A) is designed to measure the presence and intensity of dysfunctional attitudes. The higher the score, the more dysfunctional attitudes are characteristic of an individual. The 17 items are divided into two subscales: perfectionism and dependency. Each item is scored on a Likert scale from 1 to 7. Sum scores for either subscale are calculated by simple addition (32). All questionnaires employed in the survey have been validated and extensively used in international literature (33–36).

The Support Dimension Scale (SDS) consists of 14 items assessing how much a person can rely on social support in difficult life situations. Respondents are required to mark their answers on a four-point Likert scale (0-3). The following individuals and groups are included in the inventory as possible sources of social support: parent, child, spouse, partner, relative, friend, schoolmate, coworker, neighbor, helper profession, church group, association, and civic group. By using this questionnaire, the extent and strength of social relationships can be mapped. Total scores are calculated by adding up the values associated with each item. A higher total score indicates that the person feels they can rely more on members of their social environment. The Hungarian adaptation of this measurement tool was conducted by Rózsa et al., and the applicability of the shortened version has been validated by several Hungarian studies (37–40).

### 2.5 Statistical analysis

Statistical analysis was performed using IBM SPSS 26.0 and GraphPad Prism 4.00 for Windows software (GraphPad Software, San Diego, CA, USA, http://graphpad.com). Data are described by the mean, median, standard deviation, frequencies and percentages. Categorical variables were compared between groups using chi-squared or Fisher’s exact test as appropriate. Continuous variables were evaluated by Mann-Whitney test and ANOVA or Kruskal-Wallis test. Spearman’s correlations were used measuring the relationship between two variables. A paired samples t-test was conducted for comparisons and significance testing on overall average scores of cognitive function domains at the specified timepoints. Multiple/binary logistic regression (enter and forward Likelihood Ratio methods) was performed among HL survivors to identify predictors of cognitive dysfunctions. Odds ratios (OR) with 95% confidence intervals (CI) were estimated for the logistic regression models. Significance level was set at p<0.05.

Since no Hungarian control group was available, the participants’ Z-scores of all CANTAB subtests were calculated from median scores based on the normative database of 3000 healthy volunteers. The index scores of the patients and those of the normative database were compared using one-tailed non-parametric t-test. We considered a test result was impaired when a patient scored worse than the normal population by 1.5 standard deviations (SD). A domain (visual memory, attention, working memory and planning) was impaired when a subject scored ≤1.5 SD below the expected norm in an NP test with at least one significant representative parameter (11).

## 3 Results

### 3.1 Description of the sample

A total of 30 adult treatment-naive HL patients were recruited to the longitudinal study (16/14 female/male, 54/46%). The mean age at diagnosis was 35 (20-69) years. All participants were diagnosed with HL and received combination chemotherapy as per standard of care **Table 1**.

**Table 1:** The baseline characteristics of Hodgkin-lymphoma (HL) patients.

	All patients (n:30)
Median age at T1 = diagnosis time (years)	33 (20-69)
Median age at T2 (years)	33 (21-63)
Median age at T3 (years)	40 (29-70)
Time elapsed T1-T2 (months)	7 (5-13)
Time elapsed T1-T3 (years)	8,6 (0-11)
Male	14 (46%)
Female	16 (54%)
Place of permanent residence	
Village	4 (13%)
City	26 (87%)
Education level	
High school or below	22 (73%)
College or above	8 (27%)
Active employment status	22 (73%)
Inactive empolyment status	8 (27%)
HL stage	
I	0
II	6 (20%)
III	5 (16%)
IV	19 (64%)
Bulk disease	11 (36%)
Without bulk	19 (64%)
B symptom	19 (64%)
No B symptom	11 (36%)
ECOG performance status	
0	29 (96%)
1	1 (4%)
Comorbidity	6 (20%)
No comorbidity	24 (80%)
Treatment	
ABVD	22 (73%)
A+AVD	8 (27%)
Irradiation	12 (40%)
No irradiation	18 (60%)
Primary treatment response	
Complete remission	26 (87%)
Partial remission	2 (6,5%)
Stable disease	2 (6,5%)
Treatment-related side effect	
Yes	9 (30%)
No	21 (70%)
Relapse / No response	4 (13%)
No relapse	26 (86%)

B symptoms: fever, night sweats and unintentional weight loss; ECOG PS, Eastern Cooperative Oncology Group performance status; ABVD, adriamycin, bleomycin, vinblastin and dacarbazin; A+AVD, brentuximab vedotin, doxorubicin, vinblastin, dacarbazin.

### 3.2 Assessment of neurocognitive functions

**Figure 2:**
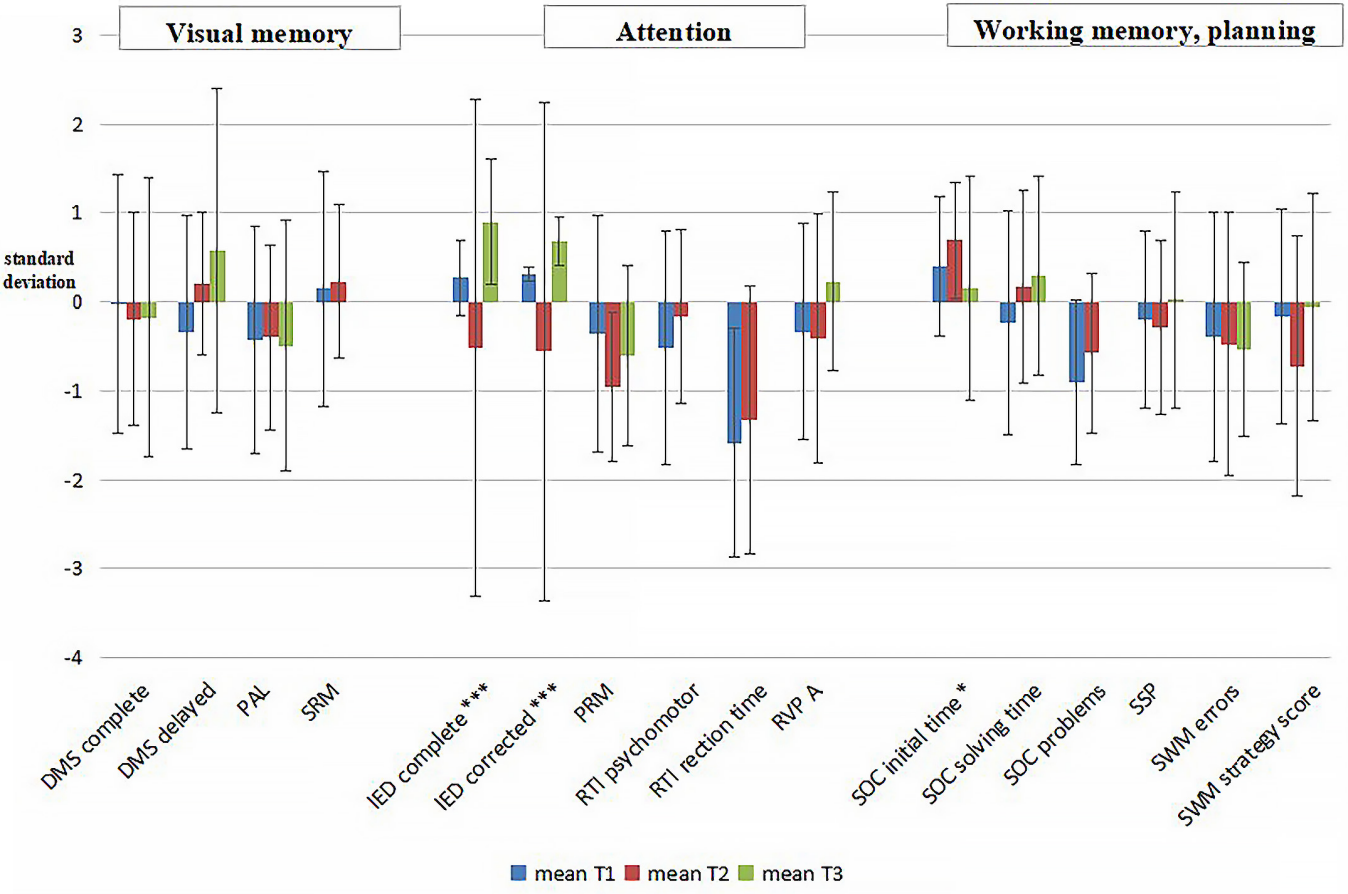
Differences between HL patients and normative CANTAB data (T1-blue n=30, T2-brown n=26, T3-green n=19). Bar - standard deviation, column - average. DMS, simultaneous and delayed matching; PAL, paired association learning; SRM, spatial recognition memory; IED, intra/extra dimensional shift; PRM, pattern recognition memory; RTI, reaction time; RVP, rapid visual information processing; SOC, stocking of Cambridge; SSP, spatial span; SWM, spatial working memory; *, p<0.05; ***, p<0.001.

The pattern of median Z-scores of the CANTAB tests and their difference from the control scores measured in subjects with HL before, immediately after treatment and at the end of the 8.6 year-follow-up are presented in **Figure 2**.

### 3.3 Pre-treatment cognitive function (T1)

**Figure 3:**
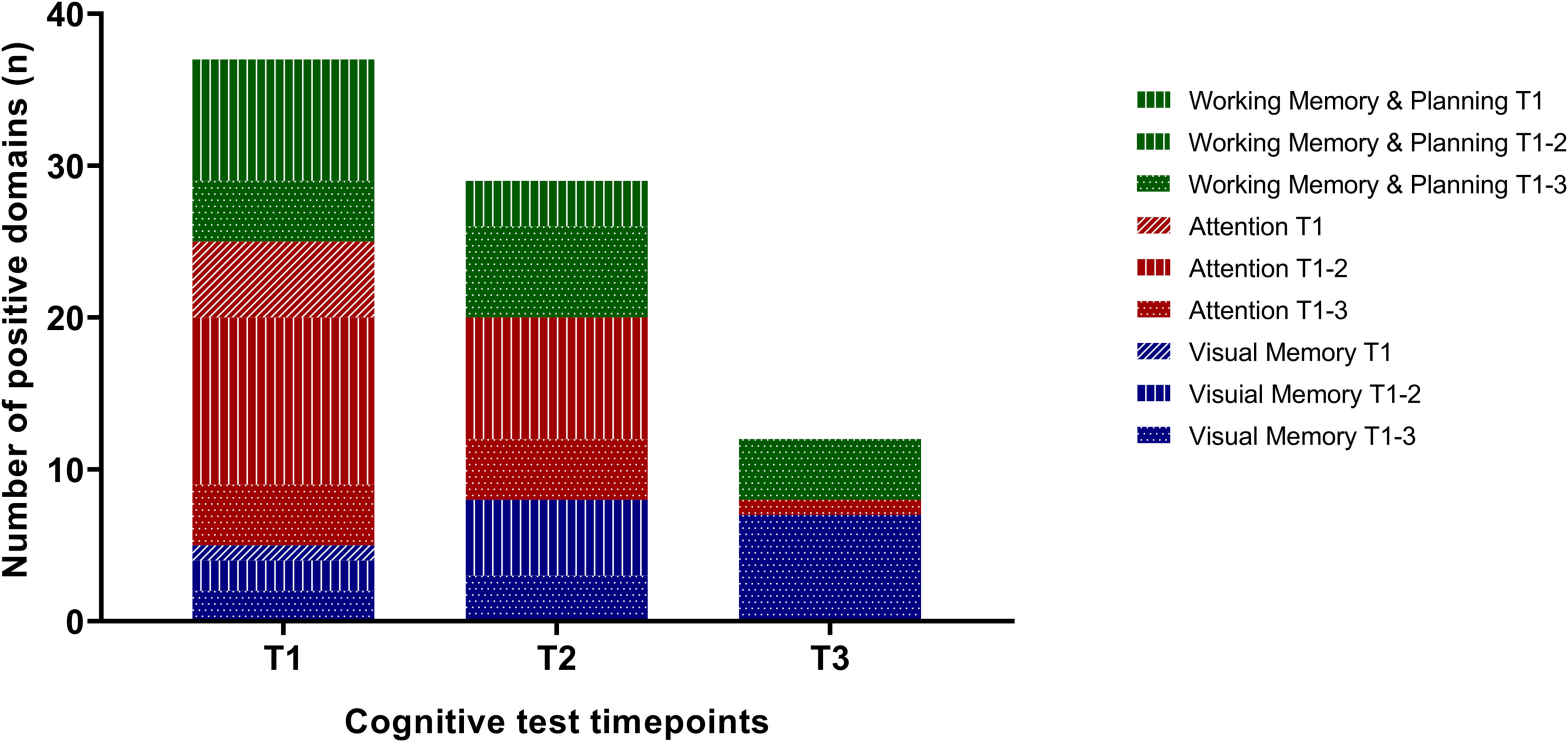
Impaired cognitive functions before (T1), immediately after treatment (T2) and at the end of the study (T3) (T1 n=30, T2 n=26, T3 n=19) based on the visual memory, attention and working memory/planning group.

77% (n=23) of all pre-treatment patients (n=30) showed cognitive impairment based on at least one positive subtest. Attention was impaired in 53% (16/30) of patients. Working memory and planning were damaged in 40% (12/30), while visual memory was affected in 17% (5/30 patients). One domain (1-visual memory, 9-attention, 4-working memory and planning) was found positive in 14 patients (47%, 14/30), two domains (1- visual memory/attention, 2- visual memory/working memory and planning, 5- attention/working memory and planning) in 8 patients (26%, 8/30) and three in 1 patient (3%, 1/30) **Figure 3** and **Supplementary Figure 1**.

As a next step, we investigated whether there are associations between positive neurocognitive subtest results and patient-related factors, specific signs and symptoms of their diseases, or therapeutic factors (after treatment period).

In terms of pre-treatment cognitive assessment, attention function was the most affected (53%, 16/30 patients). Match to sample visual search (MTS) was associated with unfavorable prognostic factors [mean ± standard deviation (p)] (III-IV. stage, age, bulky -0.444 ± 1.778/favorable 0.749 ± 0.592, p=0.013), presence of stress (PSS -0.415, p=0.039), social support (SDS -0.4, p=0.035). Pattern recognition memory (PRM) showed a close association with living conditions (city 0.003 ± 1.062/village -1.011 ± 1.239 p=0.042), age (-0.415, p=0.032) and social support (SDS 0.358, p=0.05). Comorbidities (T2DM, psoriasis, HTN) have an impact on attention function *(*0.056 ± 0.408/no comorbidities 0.281 ± 0.415 IED, p=0.013 and -1.784 ± 1.185/no comorbidities -0.112 ± 0.98 RVP, p=0.012). Our findings suggest that attention is independent of anxiety and depression.

Working memory and planning were impaired in 40% (12/30 patients). Stocking of Cambridge (SOC) subtest showed a clear association with bulk disease (-0.597 ± 1.287/without 0.332 ± 0.942 p=0.036) and correlated with several health-related questionnaires (HADS anxiety/depression, GHQ and DAS scale). Spatial span (SSP) was strongly associated with single marital status (0.33 ± 0.724/in relationship -0.608 ± 0.799 p=0.005) and anxiety (-0.43, p=0.028).

Visual memory is the least affected domain (17%, 5/30 patient). Simultaneous and delayed matching (DMS) showed a correlation with anxiety (-0.534, p=0.005) and depression (-0.612, p=0.001) and residence (city -0.087 ± 1.236/village -1.515 ± 1.514 p=0.025). Paired association learning (PAL) test was correlated with residence status (city -0.114 ± 1.38/village -1.647 ± 1.55 p=0.003) and comorbidities (-1.698 ± 1.983/no comorbidities -0.036 ± 0.682 p=0.041). These results are presented in **Table 2**. Significant correlations between cognitive subtest results and patient-related factors at timepoint 1 (T1) are shown in the **Supplementary Table 2**. Mann-Whitney test was used to establish statistical significance.

**Table 2:** Significant correlations between cognitive subtest results and health-related questionnaires at timepoint 1 (T1).

T1		HADS anxiety	HADS depression	GHQ-12	DAS-A	SOC	PSS	SDS
		Spearman 's correlation						
Domains								
Visual memory	DMS	R= - 0,534 P=0,005	R= - 0,612 P=0,001					
									SRM			R= - 0,47 P=0,015				
	PAL							
	Working memory, planning	SOC	R= - 0,406 P=0,04	R= - 0,409 P=0,038	R= - 0,407 P=0,039	R= - 0,406 P=0,04			
SSP		R= - 0.43 P=0,028						
SWM					R= - 0,452 P=0,02			R=0,42 P=0,021
Attention	MTS						R= - 0,415 P=0,039	R= - 0,4 P=0,035
	PRM							R=0,358 P=0,05
	IED							
	RTI							
	RVP							

Spearman’s rank correlation coefficient was used to establish statistical significance. DMS, Simultaneous and Delayed Matching; PAL, Paired Association Learning; SRM, Spatial Recognition Memory; IED, Intra/Extra Dimensional Shift; PRM, Pattern Recognition Memory; RTI, Reaction Time; RVP, Rapid Visual Information Processing; SOC, Stocking of Cambridge; SSP, Spatial Span; SWM, Spatial Working Memory; HADS, Hospital Anxiety and Depression scale; GHQ, General Health Questionnaire; PSS-4, Perceived Stress Scale 4; SOC-13, Sense of Coherence Scale; DAS-A, Dysfunctional Attitude Scale from A; SDS, Support Dimension Scale. Blue color indicates a negative, orange color indicates a positive correlation.

### 3.4 Post-treatment cognitive function (T2)

Cognitive impairment was observed in 77% post-treatment patients (20/26). One domain was found positive in 13 patients (50%, 13/26), two domains in 5 patients (19%, 5/26) and three domains in 2 patients (1%, 2/26).

Attention was impaired in 46% (12/26) of patients. PRM was negatively associated with anxiety (-0.402, p=0.042) and DAS scale (-0.442, p=0.027). Reaction time (RTI) depends on the living situation (city -0.814 ± 1.105/village -2.296 ± 1.501 p=0.027). Intra/extra Dimensional Shift (IED) has a positive correlation with anxiety (0.397, p=0.045), age (-0.537, p=0.005) and sense of coherence (0.42, p=0.033). Comorbidities have an impact on attention function (-0.482 ± 2.666/no comorbidities 0.36 ± 0.024 IED, p=0.013 and -0.091 ± 1.125/no comorbidities -1.802 ± 1.358 RVP, p=0.012).

Working memory and planning were impaired in 34% (9/26). The SOC subtest showed clear association with inactive employment status (-0.104 ± 0.962/active 0.449 ± 1.02 p=0.032), and correlated with several health-related questionnaires (HADS anxiety/depression, and SOC-13 scale). Spatial span (SSP) is strongly associated with living conditions (city 0.01 ± 0.846/village -1.027 ± 0.732 p=0.016), and anxiety (-0.553, p=0.003).

Visual memory was affected in 31% (8/26 patients). DMS showed a correlation with anxiety (-0.587, p=0.002), depression (-0.514, p=0.007) general health (-0.442, p=0.019) and inactive employment status (-1.431 ± 1.535/active 0.34 ± 0.972 p=0.001). The PAL test was correlated with residence status (city 0.061 ± 0.642/village -1.15/1.476 p=0.047) and comorbidities (0.002 ± 0.703/no comorbidities -1.63 ± 1.56 p=0.041). These results are presented in **Table 3**. Significant correlations between cognitive subtest results and patient-related factors at timepoint 2 (T2) are shown in the **Supplementary Table 3**. Mann-Whitney test was used to establish statistical significance.

**Table 3:** Significant correlations between cognitive functions and health-related questionnaires at timepoint 2 (T2).

T2		HADS anxiety	HADS depression	GHQ-12	DAS-A	SOC	PSS-4	SDS
		Spearman 's correlation						
Domains								
Visual memory	DMS	R= - 0.587 P=0.002	R= - 0.514 P=0.007	R= - 0.442 P=0.019				
	SRM	R= - 0.416	R= - 0.451					
		P=0.034	P=0.021					
	PAL							
Working memory, planning	SOC	R= - 0.403 P=0.04	R= - 0.451 P=0.021			R= - 0.428 P=0.029		
	SSP	R= - 0.553 P=0.003						
									SWM	R= - 0.442 P=0.024			R= - 0.542 P=0.004			
	Attention	MTS							
PRM		R= - 0.402 P=0.042			R= - 0.442 P=0.027			
IED		R=0.397 P=0.045				R=0.42 P=0.033		
RTI					R=0.451 P=0.024			
RVP								

Spearman’s rank correlation coefficient was used to establish statistical significance. DMS, Simultaneous and Delayed Matching; PAL, Paired Association Learning; SRM, Spatial Recognition Memory; IED, Intra/Extra Dimensional Shift; PRM, Pattern Recognition Memory; RTI, Reaction Time; RVP, Rapid Visual Information Processing; SOC, Stocking of Cambridge; SSP, Spatial Span; SWM, Spatial Working Memory; HADS, Hospital Anxiety and Depression scale; GHQ, General Health Questionnaire; PSS-4, Perceived Stress Scale 4; SOC ***-13***, Sense of Coherence Scale; DAS-A, Dysfunctional Attitude Scale from A; SDS, Support Dimension Scale. Blue color indicates a negative, and orange color indicates a positive correlation.

### 3.5 Correlation between cognitive function and cumulative dose of treatment

The cumulative dose of brentuximab vedotin showed a positive correlation with the PAL (0.75, p=0.05), SSP (0.857, p=0.014) and PRM (0.857, p=0.014) subtests. Similarly, the cumulative dose of bleomycin, which was associated with SOC (0.486, p=0.03). These data are presented in **Table 4**.

**Table 4:** Significant correlations between cognitive functions and cumulative doses of various chemotherapeutic agents.

T2		Cumulative dose				
		Brentuximab vedotin	Adriamycin	Bleomycin	Vinblastin	Dacarbazin
		Spearman 's correlation				
Domains						
Visual memory	DMS					
	SRM					
	PAL	R=0,75 P=0,05				
Working memory, planning	SOC			R=0,486 P=0,03		
	SSP	R=0,857 P=0,014				
	SWM					
Attention	MTS					
	PRM	R=0,857 P=0,014				
	IED					
	RTI					
	RVP					

DMS, Simultaneous and Delayed Matching; PAL, Paired Association Learning; SRM, Spatial Recognition Memory; IED, Intra/Extra Dimensional Shift; PRM, Pattern Recognition Memory; RTI, Reaction Time; RVP, Rapid Visual Information Processing; SOC, Stocking of Cambridge; SSP, Spatial Span; SWM, Spatial Working Memory.

### 3.6 Correlation between pre-treatment and post-treatment results

The paired samples T-test (n=26) suggests almost significant improvement in the working memory, planning domain between T1 and T2 (-0.192, p=0.057). At the end of the treatment, there is an improvement in anxiety (1.5, p=0.008), depression (1, p=0.07), while the social support (SDS scale) is decreased (11.692, p=0.0001). The results of the correlation between pre- and post-treatment measurements are shown in **Table 5**.

**Table 5:** Significant correlations between T1-T2 cognitive test results and health-related questionnaires.

T1 - T2	Paired samples T-test	
	Mean ± SD	P
**Working memory, planning**	-0.192 ± 0.491	0.057
**SOC**	-0.328 ± 0.691	0.023
**HADS anxiety**	1.5 ± 2.657	0.008
**HADS depression**	1 ± 2.698	0.07
**GHQ**	4.615 ± 8.198	0.008
**SDS**	11.692 ± 7.719	0.0001

SOC, Stockings of Cambridge; HADS, Hospital Anxiety and Depression scale; GHQ, General Health Questionnaire; SDS, Support Dimension Scale.

### 3.7 Follow-up cognitive function (T3)

During follow-up (8.6 years on average), limiting factors have emerged. Among the subdomains of cognitive function, the new test bank did not include RTI psychomotor/reaction time, SOC, SRM and MTS. As can be seen, these tests were most recently used to assess attention function. Cognitive dysfunctions were detected in 52% (10/19 patients) of the HL cases.

In the visual memory domain, 36% of patients (7 out of 19) exhibited impairments in DMS, which was significantly affected by an unfavorable prognosis (-1.154 ± 0.949/favorable 0.913 ± 1.395 p=0.01).

Working memory and planning were involved in 21% (4/19 patients). Unfavorable prognosis (-0.346 ± 0.765/favorable 0.995 ± 1.047 p=0.003) was also found to significantly influence SOC, which was also negatively associated with the sense of coherence (-0.451, p=0.051). Additionally, SSP showed a correlation with anxiety (-0.665, p=0.002), depression (-0,462, p=0.046) general health (-0.588, p=0.008), DAS scale (-0.527, p=0.02) and living conditions (city 0.433 ± 1.102/village -0.23 ± 0.68 p=0.003).

Given these data, it is not surprising that attention function was the least affected (5%) during the follow-up period. It was associated with the presence of stress (IED, 0.467, p=0.04), and education (graduation 0.616 ± 0.267/postgraduation 0.922 ± 0.154 IED, p=0.027). The result of the follow-up period (T3) is presented in **Table 6**.

**Table 6:** Significant correlations between cognitive subtest results and health-related questionnaires at T3.

T3		HADS anxiety	HADS depression	GHQ-12	DAS-A	SOC	PSS-4	SDS
		Spearman 's correlation						
Domains								
**Visual memory**	DMS							
	SRM							
	PAL							
**Working memory, planning**	SOC					R = - 0.453 P=0.051		
	SSP	R= - 0.665 P=0.002	R= - 0.462 P=0.046	R= - 0.588 P=0.008	P= - 0.527 P=0.02			
	SWM		R= - 0.612 P=0.005		R= - 0.443 P=0.057			
**Attention**	MTS							
	PRM							
	IED						R=0.476 P=0.04	
	RTI							
	RVP							

Spearman’s rank correlation coefficient was used to establish statistical significance. DMS, Simultaneous and Delayed Matching; PAL, Paired Association Learning; SRM, Spatial Recognition Memory; IED, Intra/Extra Dimensional Shift; PRM, Pattern Recognition Memory; RTI, Reaction Time; RVP, Rapid Visual Information Processing; SOC, Stocking of Cambridge; SSP, Spatial Span; SWM, Spatial Working Memory; HADS, Hospital Anxiety and Depression scale; GHQ, General Health Questionnaire; PSS-4, Perceived Stress Scale; SOC ***-13***, Sense of Coherence Scale; DAS-A, Dysfunctional Attitude Scale from A; SDS, Support Dimension Scale. Blue color indicates a negative, orange color indicates a positive correlation.

In the T3 period, there was a directly proportional association with the PAL test and the cumulative dose of adriamycin (0.473, p=0.041), vinblastine (0.507, p=0.027) and dacarbazin (0.507, p=0.027). (not shown) Significant correlations between cognitive subtest results and patient-related factors at timepoint 3 (T3) are shown in the **Supplementary Table 4**. Mann-Whitney test was used to establish statistical significance.

### 3.8 Longitudinal assessment of mental health questionnaires and cognitive function

Before treatment, seven patients (24%) had caseness scores with HADS anxiety and 4 (14%) with HADS depression. 8 (26%) of HL survivors had abnormal levels of distress with GHQ. Following treatment, both anxiety (3/26) and depression (n=3/26) levels decreased to 12%, GHQ reduced to 15% (4/26) while the sense of coherence and support dimension also declined. However, in the long term, anxiety persisted in 20% of the cases. Among long-term survivors, anxiety, depression, mental health, perceived stress, and dysfunctional attitude decreased, but the sense of coherence and support dimension improved. **Table 7**.

**Table 7:** Chronological distribution of the mean, standard deviation, and patients’ results as the health-related questionnaires are concerned.

	T1 (n:30)	T2 (n:26)	T3 (n:19)
	Mean ± SD		
HADS anxiety	5.83 ± 3.415	3.93 ± 4.127	5.11 ± 3.985
HADS depresison	3.37 ± 3.81	2.03 ± 3.449	2.74 ± 2.306
GHQ	24.63 ± 5.756	19.73 ± 7.961	21.74 ± 5.162
PSS-4	7.9 ± 1.398	6.9 ± 2.759	7.79 ± 1.751
DAS-A	46.8 ± 21.069	44.6 ± 21.167	53.32 ± 27.021
SOC-13	56.37 ± 7.641	52.17 ± 15.89	60.74 ± 4.736
SDS	21.87 ± 5.661	10.5 ± 7.934	13.63 ± 7.266
	Number of patients (%)		
HADS anxiety			
normal (0-7)	23 (76%)	23 (88%)	15 (80%)
borderline (8-10)	3 (10%)	0	1 (5%)
abnormal (11-21)	4 (14%)	3 (12%)	3 (15%)
HADS depression			
normal (0-7)	26 (86%)	23 (88%)	18 (95%)
borderline (8-10)	2 (7%)	1 (4%)	1 (5%)
abrnormal (11-21)	2 (7%)	2 (8%)	0
GHQ			
normal (0-4)	22 (74%)	22 (85%)	16 (85%)
abnormal (≥5)	8 (26%)	4 (15%)	3 (15%)

HADS, Hospital Anxiety and Depression scale; GHQ, General Health Questionnaire; PSS-4, Perceived Stress Scale 4; DAS-A, Dysfunctional Attitude Scale from A; SOC-13, Sense of Coherence Scale; SDS, Support Dimension Scale.

Repeated measures analysis of variance test was performed in order to assess independent determinants of 8.6-year changes in neurocognitive tests and health-related questionnaires as dependent variables. IED complete/corrected, SOC, HADS anxiety, GHQ, SOC-13 yielded significant results. In this respect, A+AVD treatment exerted combined effects with education on changes in IED complete **Supplementary Table 5**.

## 4 Discussion

Considering the successes in treating HL and minimizing the short- and long-term reversible and irreversible treatment-related side effects, the reintegration of young, recovered patients back into their daily lives should receive a high priority. The average age at which HL is diagnosed tends to be around 40 years, a time when individuals are often at the peak of their careers. Recognizing this disease’s substantial social and economic importance is crucial (41, 42). In the current study, we found cognitive impairment in 77% of patients at baseline, 77% after receiving first line therapy and 52% after follow-up period. Cognitive impairment has been reported in 13-70% of patients receiving chemotherapy (solid cancer/hematological malignancies), and persistent impairment was observed in about 15-35% during follow-up (40, 41). Multifactorial aetiological factors are involved in the development of CRCI, although the mechanisms are still not completely understood. According to the literature data, multiple factors, including genetic predisposition, age, psychological, social, and demographic factors, can increase the risk of CRCI. Data on CRCI show diversity in several studies. Based on literature data, this is mainly caused by the following reasons: differences in disease severity and type of treatment regimens, small sample sizes and, therefore, limited power, definitions of cognitive impairment, varied criteria for cognitive impairment cut-offs variations in measure used, varying measurement time frames to detect CRCI significantly (14, 43). There are three ways to measure CRCI. The first option is subjective self-reported questionnaires, the second way to perform an objective neuropsychological test, and the third option is brain imaging examinations, which might help to characterize the potential influencing factors. It is currently unclear how severe the cognitive impairment in Hodgkin’s lymphoma patients is at diagnosis, how it changes immediately after treatment, and how it is expected to change years after therapy. The clinical trials were primarily conducted on patients with solid cancers, with a focus on the most prevalent type, breast cancer. However, for hematological cases, the trials paid attention to survivors of acute lymphoblastic leukemia and primary central nervous system lymphoma (8, 44, 45). Limited published data available on long-term HL survivors (8, 15–17, 46). Some literature data suggested that cognitive function was already impaired among treatment-naive HL patients, as reported by previous studies (19–21).

### 4.1 Pre-treatment part

Pre-treatment patient-related factors have not been thoroughly investigated thus far. Our research found that individuals from smaller settlements exhibited impaired performance in the visual memory (DMS, PAL) and attention domain (PRM). Patients, who suffer from bulk disease, demonstrated impaired working memory and planning domain (SOC). Additionally, in male patients and advanced HL stage, attention function impairment (MTS) was observed. The study found that significant psychological distress, such as anxiety (HADS anxiety) depression (HADS depression), and general mental health (GHQ-12), was related to deficits in working memory, planning (SOC, SSP) and visual memory (DMS, SRM). Surprisingly, attention function, which was the most affected domain, did not show a significant correlation with anxiety and depression. However, attention function did correlate with unfavorable prognosis, indicating that impairment may be linked to the disease. Juríczková et al. also described cognitive impairment prior to treatment in 40 HL cases associated with the disease (21), which was also confirmed by Dan Fayette’s study (20). It can be concluded that individuals residing in smaller municipalities who are unemployed and suffering from bulk disease and unfavorable prognosis, exhibit significant cognitive impairment and increased psychological distress before treatment.

### 4.2 Post-treatment

A number of neuropsychological studies have investigated cognitive impairment following chemotherapy (8, 15–17, 46–49), but fewer have compared it with the pre-treatment state (20, 21). In our study, there was a significant improvement in Stocking of Cambridge test and also in working memory and planning between the pre- and post-treatment examination (T1-T2). A study of 242 lymphoma cases (51 of which were HL) found significant cognitive decline at verbal memory tests and delayed recall, attention and executive function between pre- and post-treatment (19). Despite the existing impairment, we noticed an improvement in anxiety, depression, mental health, perceived stress, and dysfunctional attitude following the completion of the treatment. In spite of the improvement, we found significant loss in several domains of health-related questionnaires, which were associated with neurocognitive impairment. An Italian research group described that a decline in cognitive function was connected to a lower quality of life, as both subjective and objective cognitive measures showed negative correlations with most health-related questionnaires’ scores (50). The success of cognitive rehabilitation in long-term HL survivors could provide insight into strategies for improving health-related questionnaire decline (51). Additionally, the cumulative dose of brentuximab vedotin was directly proportional with a subtest of each of the 3 main domains (attention; visual memory; working memory and planning), and the cumulative dose of bleomycin was also directly proportional with a subtest of working memory. Bleomycin (which is cardio and pulmonary toxic) has been associated with impaired visuomotor processing speed in HL female survivors, as reported by Phillips et al. (18).

In our study, majority of the HL patients (77%) had impairment in at least one cognitive domain. Attention (and processing speed) was the most affected at 46%, working memory, planning (executive function) at 34%, while visual memory (recall and learning) at 31%. A research by a team from Israel found 30% of HL survivors (51 patients) with impaired cognitive function in ≥2 domains using CANTAB. Treatment completion for HL survivors ranged from six months to 5 years (16). Mariegaard et al. published that 39% of lymphoma survivors (115 patients, 65 with HL) showed impairment on executive function tests made with self-reported cognitive questionnaires (the mean period time elapsed after the completion of treatment was 29.6 months) (46). We identified again the associations between objective neurocognitive deficits and inactive employment status. Krull et al. have previously described in childhood HL survivors that unemployed status was associated with more severe impairments in working memory and task performance (45). Kiserund et al. reported that among 281 subjects following autologous hematopoietic stem cell transplantation, 102 unemployed subjects had worse cognitive function. Erhard et al. also found cognitive impairment among unemployed childhood non-Hodgkin lymphoma survivors (187 patients) (52).

### 4.3 Follow up

Although no study has yet been done in this area (adult HL patients) with a pre-treatment -, a post-treatment - and a follow-up examination (an average follow-up of 8.6 years), the results should be treated with caution due to the many limiting factors. Among long-term survivors, anxiety, depression, mental health, perceived stress, and dysfunctional attitude decreased again, but the sense of coherence and support dimension improved. However, the most notable fact was a clear correlation between the PAL test and the cumulative dose of adriamycin, vinblastine and dacarbazine in the T3 period. CANTAB-PAL is an 8-minute non-verbal task that has been widely studied in diagnostically, culturally and linguistically diverse populations. This measure of visual associative memory has shown good reliability over time and correlation with traditional neuropsychological tests and everyday functional measures. CANTAB-PAL has shown a sensitivity of 83% and a specificity of 82% in mild cognitive impairment. CANTAB-PAL has the most validation in Alzheimer’s disease and related dementia (53, 54). Over an extended period, the results call attention to a potentially increased risk of developing secondary dementia as a result of treatment.

## 5 Conclusions

Similar to previous studies, we confirmed that cognitive impairment in adult patients with Hodgkin lymphoma is present even before treatment begins. This impairment is primarily influenced by disease-related factors, such as bulky disease and unfavorable prognostic factor, as well as patient-related factors, including the presence of comorbidities and living conditions. For the first time, we have demonstrated a significant improvement between the tests conducted immediately before the treatment and at the end of the treatment. Considering that between T1-T2 timepoints only disease-associated factors change due to the short time span, we attribute this improvement to the beneficial effect of therapy. This finding could be important for clinical practice, as patients experiencing cognitive impairment at the time of diagnosis can be reassured that treatment may improve their symptoms. In the long run, this improvement could enhance not only mental health but also physical well-being. Since cognitive functions and, consequently, quality of life can be improved, it is essential to provide proper care for these groups from the beginning of treatment and to implement appropriate therapeutic strategies in a timely manner. Our study is not without limitations. The main limitations of this research were: small sample size, single institution, the lack of a matched healthy control population, - some neurocognitive test (RTI psychomotor/reaction time, SOC, SRM and MTS) at timepoint 3 and - neuroimaging examination. Further, longitudinal clinical studies with more extensive patient size could help characterize the objective neurocognitive outcomes of HL survivors. Our results draw attention to the fact that pre-treatment cognitive impairment is a real-life problem among treatment-naive HL patients, similar to other malignant diseases.

## Data Availability

The original contributions presented in the study are included in the article/**Supplementary Material**. Further inquiries can be directed to the corresponding author.
