# Supplementary figures and images for: Longitudinal study of cognitive and mental functions among adult Hodgkin-lymphoma survivors, based on data from a primary treatment center in Hungary
Source: Front Oncol. 2025 Feb 17;15:1509424. doi: 10.3389/fonc.2025.1509424 (PMC11872912; doi:10.3389/fonc.2025.1509424)

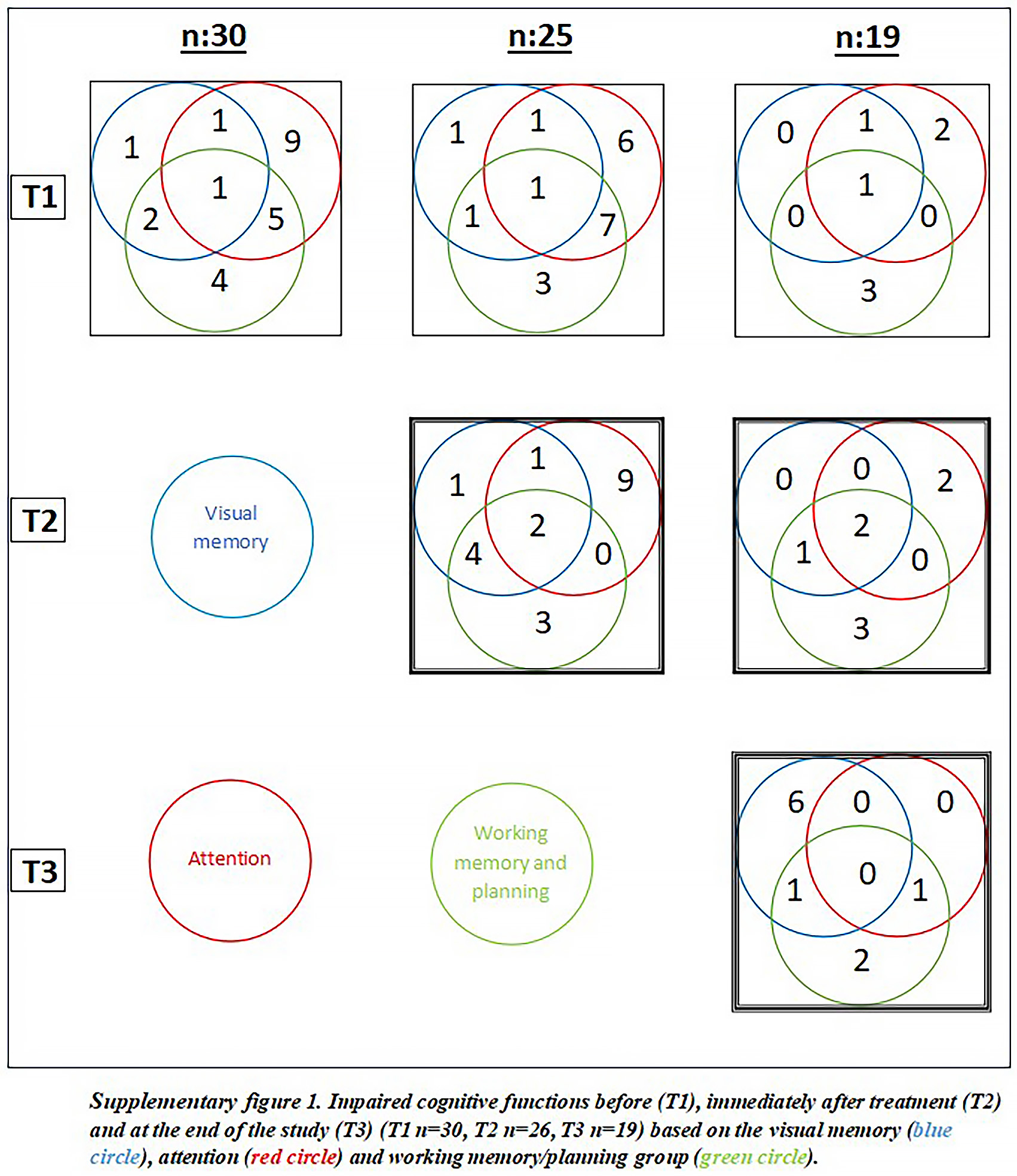

Supplement: Supplementary file 1 [file Image1.jpeg]

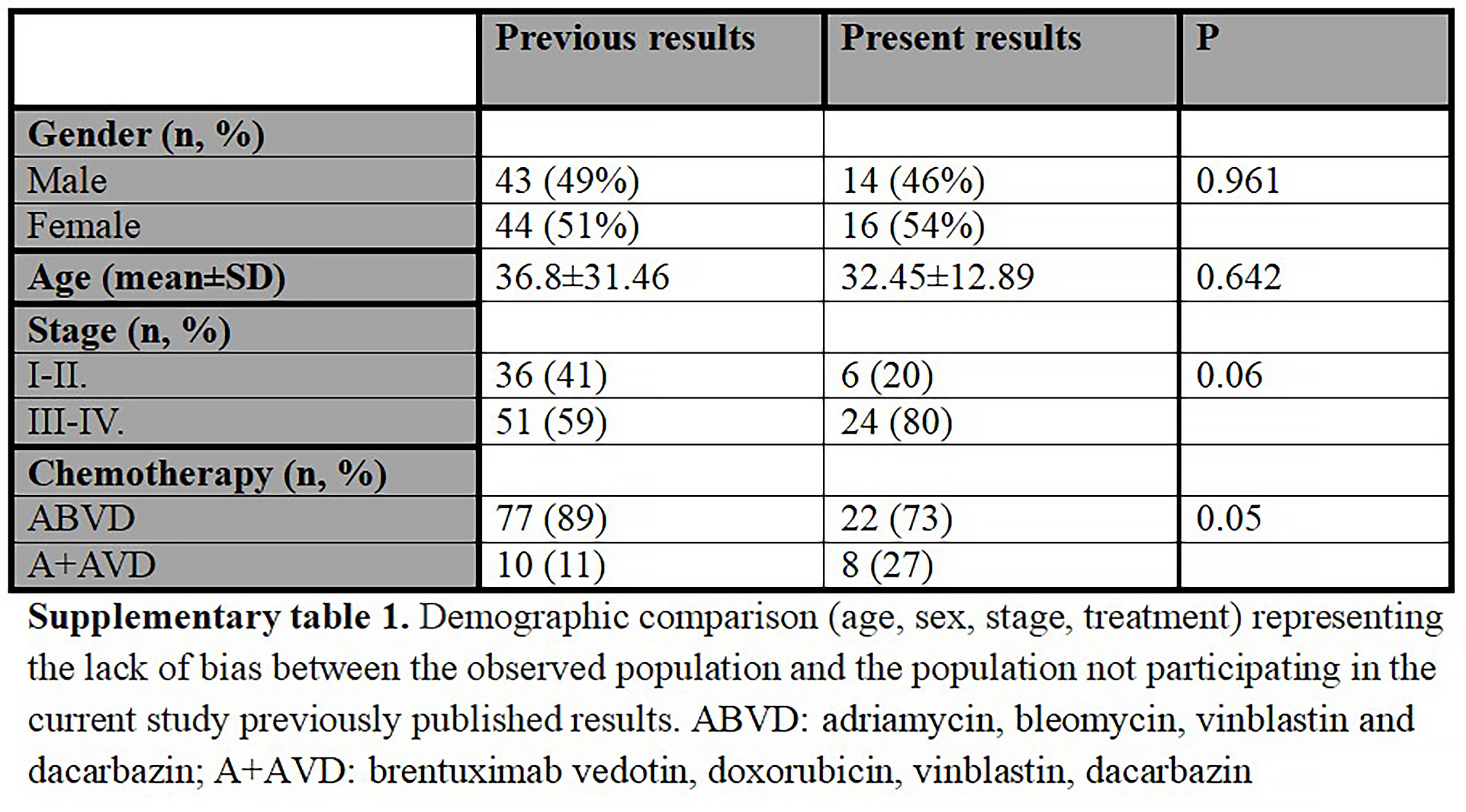

Supplement: Supplementary file 2 [file Supplementaryfile1.jpeg]

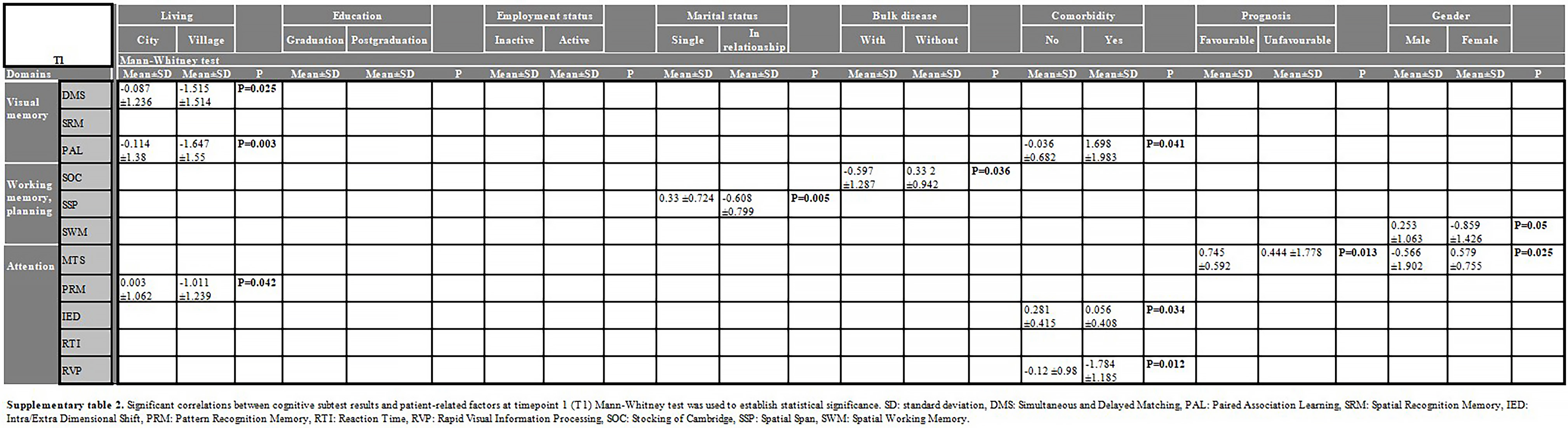

Supplement: Supplementary file 3 [file Supplementaryfile2.jpeg]

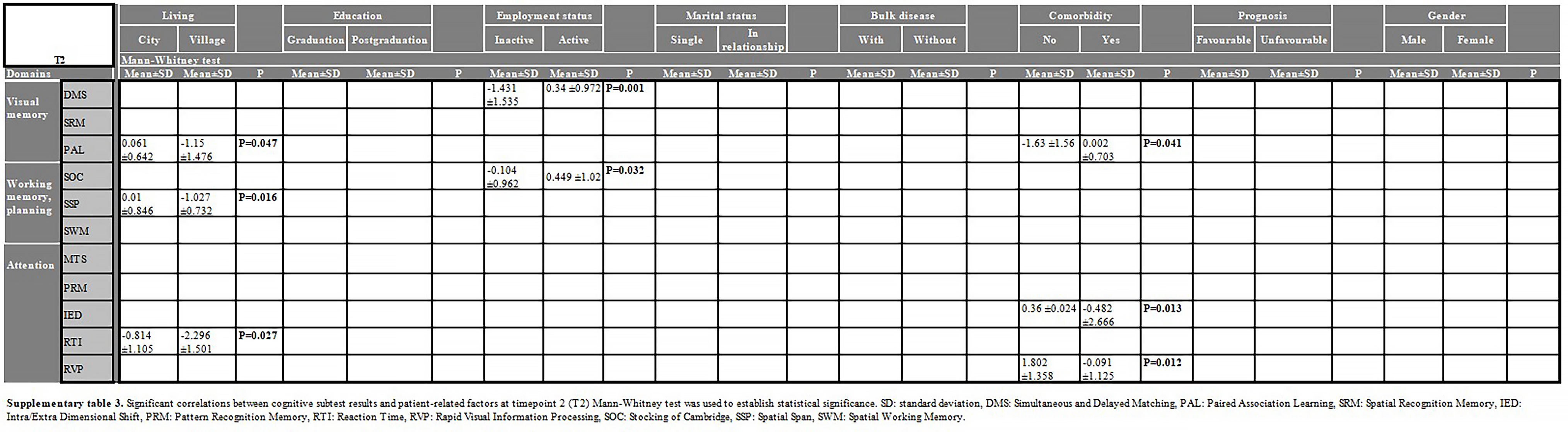

Supplement: Supplementary file 4 [file Supplementaryfile3.jpeg]

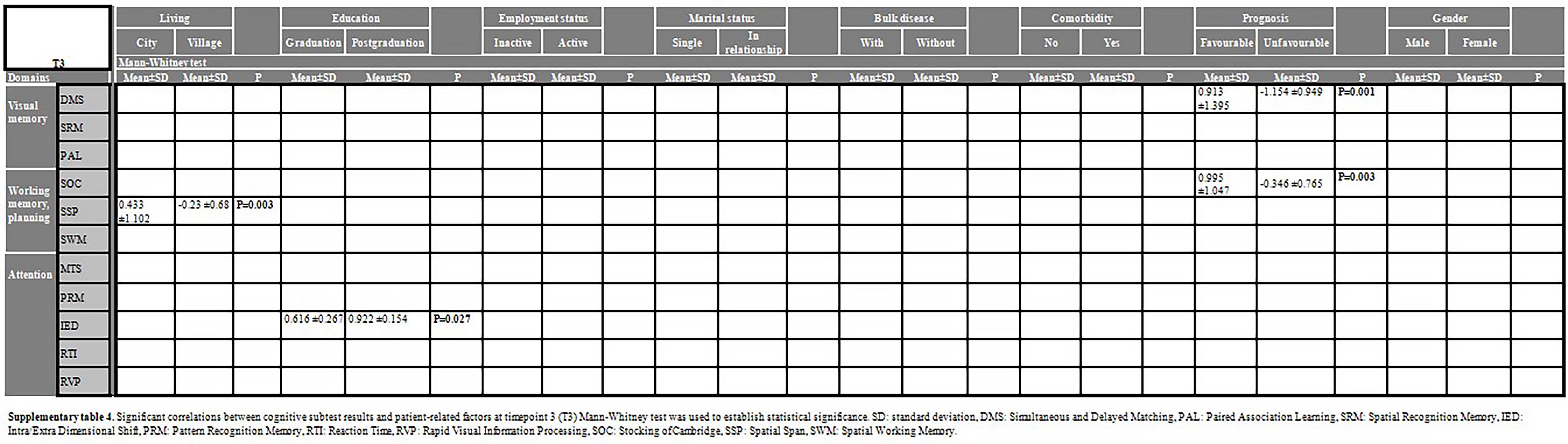

Supplement: Supplementary file 5 [file Supplementaryfile4.jpeg]

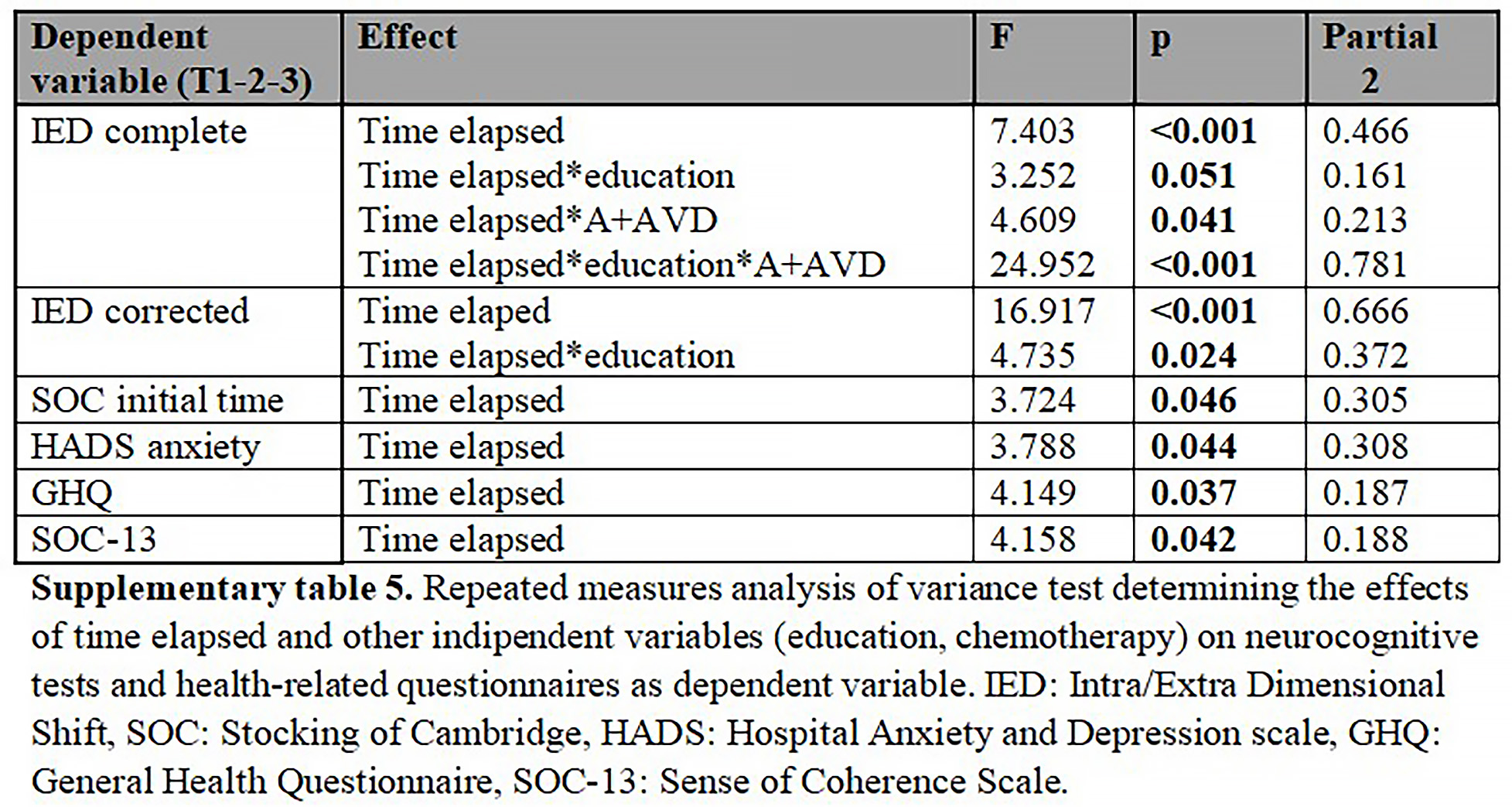

Supplement: Supplementary file 6 [file Supplementaryfile5.jpeg]
